# Health state utilities associated with major clinical events in the context of secondary hyperparathyroidism and chronic kidney disease requiring dialysis

**DOI:** 10.1186/s12955-015-0266-9

**Published:** 2015-06-30

**Authors:** Evan W. Davies, Louis S. Matza, Gavin Worth, David H. Feeny, Jacqueline Kostelec, Steven Soroka, David Mendelssohn, Philip McFarlane, Vasily Belozeroff

**Affiliations:** Outcomes Research, Evidera, W6 8DL London, UK; Outcomes Research, Evidera, Bethesda, MD USA; Amgen (Europe) GmbH, Zug, Switzerland; Department of Economics, McMaster University, Hamilton, ON Canada; Department of Medicine, Dalhousie University, Halifax, NS Canada; Department of Nephrology, Humber River Hospital, Toronto, ON Canada; Division of Nephrology, St. Michael’s Hospital, Toronto, ON Canada; Amgen, Inc, Thousand Oaks, CA USA

**Keywords:** Utility, Chronic kidney disease, Secondary hyperparathyroidism, End-stage renal disease, Standard gamble

## Abstract

**Background:**

Patients with chronic kidney disease (CKD) and secondary hyperparathyroidism (SHPT) who require dialysis are at increased risk for cardiovascular events and bone fractures. To assist in economic evaluations, this study aimed to estimate the disutility of these events beyond the impact of CKD and SHPT.

**Methods:**

A basic one-year health state was developed describing CKD and SHPT requiring dialysis. Further health states added acute events (cardiovascular events, fractures, and surgical procedures) or chronic post-event effects. Acute health states described a year including an event, and chronic health states described a year subsequent to an event. General population participants in Canada completed time trade-off interviews from which utilities were derived. Pairwise comparisons were made between the basic state and event, and between comparable health states.

**Results:**

A total of 199 participants (54.8% female; mean age = 46.3 years) completed interviews. Each health state had ≥130 valuations. The mean (SD) utility of the basic health state was 0.60 (0.34). For acute events, mean utility differences versus the basic state were: myocardial infarction, −0.06; unstable angina, −0.05; peripheral vascular disease (PVD) with amputation, −0.33; PVD without amputation, −0.11; heart failure, −0.14; stroke, −0.30; hip fracture, −0.14; arm fracture, −0.04; parathyroidectomy, +0.02; kidney transplant, +0.06. Disutilities for chronic health states were: stable angina, −0.09; stroke, −0.27; PVD with amputation, −0.30; PVD without amputation, −0.12; heart failure, −0.14.

**Conclusions:**

Cardiovascular events and fractures were associated with lower utility scores, suggesting a perceived decrease in quality of life beyond the impact of CKD and SHPT.

**Electronic supplementary material:**

The online version of this article (doi:10.1186/s12955-015-0266-9) contains supplementary material, which is available to authorized users.

## Background

Chronic kidney disease (CKD) involves a gradual and usually permanent loss of kidney function. The final stage, end-stage renal disease (ESRD; Stage 5), is characterized by kidney failure and the requirement for dialysis [1–3]. Most stage 5 CKD patients have abnormal blood mineral levels and elevated levels of parathyroid hormone (PTH), a condition known as secondary hyperparathyroidism (SHPT) [4–6]. Patients with CKD and SHPT typically experience a wide range of symptoms such as bone and joint pain, as well as psychological symptoms that are associated with lower health-related quality of life [7–11]. In patients with CKD, SHPT has been found to be associated with increased resource utilization [12], treatment costs [13], and mortality [14, 15].

CKD and SHPT are also associated with increased risk of serious cardiovascular and bone-related complications. Cardiovascular disease is the leading cause of death and disability in this population [16–21]. Elevated PTH, calcium, and phosphorus levels are associated with increased risk of cardiovascular events and cardiovascular-related death in patients with CKD [18]. Sustained control of these biomarkers is associated with increased survival [22]. Furthermore, abnormal levels of PTH and other mineral metabolism indicators are associated with increased risk of bone fractures [23, 24]. Pharmacological treatments for PTH and mineral imbalances in CKD include vitamin D sterols, phosphate binders, and calcimimetic agents [2, 25–28]. When pharmacotherapy and other minimally invasive treatment options are ineffective, surgical interventions (e.g., parathyroidectomy) may be necessary [28, 29].

As new treatments are developed for patients with CKD and SHPT, it is important to evaluate their cost-effectiveness to demonstrate their value to clinicians and third-party payers. Many of the cost-effectiveness analyses of CKD treatments are cost-utility models, which incorporate the preferences of individuals for various health states and treatment-related outcomes [5, 30–33]. In these models, treatment outcome is quantified in terms of quality-adjusted life years (QALYs), which combine duration of life and quality of life into a single metric. The quality of life component is based on utilities, which are values representing the strength of preferences for health states, anchored on a scale with 1 representing full health and 0 representing dead [34, 35].

Although utility values for CKD have been published [36, 37], little is known about the utility or disutility (i.e., utility decrease) associated with cardiovascular events and bone fractures in patients with CKD and SHPT. Consequently, modelers have often had to use utility data derived from other populations such as patients with osteoporosis or cardiovascular disease without CKD [30, 32, 33, 38], leading to considerable uncertainty in the resulting cost-utility estimates. Therefore, the objective of the current study was to obtain utility values associated with cardiovascular events and fractures in the context of SHPT and CKD requiring dialysis. In addition, utilities were obtained for parathyroidectomy and kidney transplant, the two surgical procedures that are common in this population.

## Methods

### Health state development

Health state descriptions were drafted based on patient interviews, interviews with healthcare providers, and literature review. Qualitative research was conducted with 54 patients diagnosed with CKD and SHPT who were receiving hemodialysis. These interviews elicited descriptions of patients’ experience with CKD and SHPT, including symptoms and impact. Information obtained from the patient interviews was used when drafting the structured interview guides for subsequent clinician interviews.

Telephone interviews were conducted with three Canadian-based physicians (co-authors DM, PM, and SS) who specialize in treatment of patients with CKD and SHPT as well as related cardiovascular events and fractures. Interviews were first conducted to inform health state development, with questions focusing on symptoms and impact of CKD and SHPT on a patient’s life, as well as the impact of dialysis treatment. Physicians also answered questions about cardiovascular events and fractures within this patient population, as well as differences or similarities with these events in non-CKD patients. Health states were subsequently drafted and then reviewed by the physicians for clarity and accuracy.

Literature was reviewed to inform the clinician interview questions and ensure that the health state descriptions were consistent with published research. Literature searches focused on CKD and SHPT [4, 8, 39]; cardiovascular events in the context of CKD and SHPT [40–47]; fractures in the context of CKD and SHPT [4, 5]; and surgical interventions for patients with CKD and SHPT [5, 39, 48].

Health states were tested in a pilot study conducted with 19 general population participants (13 female; mean age = 31.6 years; age range = 18 to 48) recruited through an online advertisement. Each participant valued the health states using both time-trade off (TTO) and standard gamble (SG) methods [49] in order to determine which of the methods was most appropriate for the time horizon of the task (one year). The order in which participants completed the two tasks was randomized (11 completed SG first; eight completed TTO first). Both TTO and SG methods yielded logical data (i.e., utilities in a reasonable range, with logical discrimination among health states). After completing the TTO and SG tasks to rate the health states, participants answered a series of qualitative questions designed to identify any issues with the rating tasks and health states. Although the TTO and SG methods elicited similar results, participants reported that the TTO was easier to understand and complete. Subsequently, the TTO method with a one-year time horizon was used in the main study. Following feedback from pilot study participants, minor edits to health states were made to improve clarity and ease of comprehension.

### Final health states administered in time trade-off interviews

The final set of health state descriptions included a “basic health state” (health state A), which was designed to represent a patient with CKD and SHPT on dialysis. This health state included statements in four categories: a description of CKD with SHPT, symptoms, impact, and dialysis. The full text of this health state and all other health states is presented in Additional file 1. An Additional 15 “event health states” (health states B–P) included this basic health state, plus the addition of statements describing a cardiovascular event, a fracture, or a surgical procedure. Every health state was presented to participants as lasting for a single hypothetical year.

Eleven of the additional health states represented cardiovascular events (B–G and L–P). Because some of the cardiovascular events are likely to have long-term effects that are different from the acute experience of the event, both *acute* and *chronic* health states were developed. The acute health states described a year in which the event occurred, beginning with the event itself followed by ongoing consequences for the remainder of the year. The chronic health states described the ongoing functioning of a patient who had experienced one of these events in a previous year. These chronic health states can be characterized as *post-event health states* that describe the chronic ongoing impact of an event that occurred in the past, instead of the acute impact of the event itself.

Six acute health states (B–G) described acute cardiovascular events: myocardial infarction, unstable angina, heart failure exacerbation, peripheral vascular disease (PVD) with amputation, PVD without amputation, and stroke. Five chronic health states (L–P) were designed to represent ongoing long-term impact of previous cardiovascular events: stable angina (which is a possible chronic condition that may follow MI or unstable angina), heart failure, PVD with amputation, PVD without amputation, and stroke. In each of these chronic health states, the timing of the event was clearly explained. For example, the chronic stroke health state (N) included the following explanation: “Prior to this year, you experienced a stroke…The continuing effects on your life include the following symptoms and impact”.

Two health states described a fracture of the hip (H) and a fracture of the arm (I). Consistent with clinicians’ input during the interviews, the hip fracture included statements with more serious functional impairment than the arm fracture. These two fracture locations were chosen for inclusion in the health states in order to represent a broad range of the potential impact of a fracture. Two surgical procedure health states describing parathyroidectomy (J) and kidney transplant (K) were also included to represent the potential surgical treatment options for this patient group. These surgical procedures were selected based on review of the literature and input from clinicians indicating that they are common treatment options for patients with CKD and SHPT on dialysis [5, 39, 48, 50–52]. Parathyroidectomy was considered important for the current set of health states because it is directly related to the mineral imbalances associated with SHPT.

### Participants

All participants were required to be (1) at least 18 years old; (2) able to understand the assessment procedures; (3) able and willing to give written informed consent; and (4) residing in Canada. Inclusion criteria did not specify particular clinical characteristics because interviews were intended to yield utilities that may be used in cost-utility analyses for submission to reimbursement authorities, most of whom prefer utilities that represent general population values [53–56]. Participants were recruited via local newspaper and online advertisements in Toronto, Canada.

### Time trade-off utility interview procedures and scoring

Utilities were derived by eliciting values for the health state descriptions in a TTO utility interview with a one-year time horizon conducted by a single interviewer. The one-year time horizon was used so that the interviews could capture the impact of relatively brief health-related events. Each participant rated the basic health state (A) describing CKD and SHPT with dialysis, followed by 10 of the 15 additional health states (B–P). The 10 event health states to be rated by each participant were determined by block randomization. To introduce participants to the health state descriptions, a ranking exercise was conducted prior to TTO utility elicitation. Health states were presented in random order on individual cards, and each participant ranked the health states in order of preference.

After completing the introductory ranking task, health state utilities were obtained using the TTO method. For each health state, participants were offered a choice between spending a one-year period in this health state versus spending shorter amounts of time (in one-month increments) in the full health state. Choices varied by one-year increments and were presented in the following pattern: 12 months in full health, 0, 11, 1, 10, 2, 9, 3, 8, 4, 7, 5, 6. Choices were presented visually with a booklet illustrating one choice per page. Each health state rated as better than dead received a utility value on a scale with the anchors of dead (0) and full health (1). The assigned value was calculated based on the choice in which the respondent is indifferent between *y* months in the health state being evaluated and *x* months in full health (followed by *y* – *x* months dead). The resulting utility estimate (*u*) is calculated as *u* = *x*/*y*.

If participants indicated that a health state was worse than dead, the interviewer altered the task and offered a choice between immediate death (alternative 1) and a one-year life span (alternative 2) beginning with varying amounts of time in the health state being rated, followed by full health for the remainder of the one-year timeframe. For these health states, the current study used a bounded scoring approach, which is commonly used to avoid highly skewed distributions for negative utilities [49]. This scoring approach limits the utility range of health states worse than dead to values between 0 and −1. To compute these negative values, the current study used the Dolan method [57] as described by Rowen & Brazier [35]: *u* = *−x/t*, where *x* is the number of months in full health, and *t* is the total life span of alternative 2 in the TTO choice. In the current study, *t* was 12 months, which is the number of months in the health state being rated plus subsequent months in full health.

### Data collection and statistical analysis procedures

Each participant attended one interview conducted in Toronto, Canada in May 2013. Participants provided written informed consent, completed a brief demographic and clinical form, and then participated in a utility interview. Participants received remuneration of C$50 for their time. All procedures and materials were approved by an independent Institutional Review Board (Schulman Associates IRB; Protocol number 20130125). Statistical analyses were completed using SAS version 9.2 (SAS Institute, Cary, NC).

Continuous variables are summarized as means and standard deviations, and categorical variables are summarized as frequencies and percentages. The difference in utility, which may be either a disutility (i.e., utility decrease) or added utility (i.e., utility increase), associated with each health-related event or treatment modality was calculated by subtracting the utility of each event health state (B–P) from the utility of health state A. This utility difference quantifies the impact of each event on preferences for one-year health states in the context of CKD, SHPT, and dialysis. A series of independent t-tests were conducted to compare utility scores among various demographic subgroups (age, gender, religiosity).

Pairwise comparisons between health states, using t-tests, were conducted to examine utility differences between health states. T-tests were considered exploratory, and therefore no adjustments were made for multiple comparisons. The basic health state (A) was compared to each of the other health states that included a description of a cardiovascular, fracture, or surgical event (B–P). T-tests were also performed to compare between selected pairs of event health states that were conceptually comparable to each other: acute vs. chronic stroke; arm vs. hip fracture; myocardial infarction vs. unstable angina; and PVD with amputation vs. PVD without amputation. These analyses were performed to examine whether there were differences in valuations between specific pairs of comparable health states.

## Results

### Sample description

A total of 530 individuals responded to the advertisements, and 264 of these were reached for screening. Of the 264 screened participants, 260 were eligible, 226 were scheduled for interviews, and 202 attended interviews. Three of the 202 participants were unable to complete the TTO interview procedures. Thus, a total of 199 valid interviews were completed (Table 1). The sample had a mean age of 46.3 years (SD = 13.8) and was 54.8 % female. Approximately half of the sample (49.7 %) reported no health conditions, 22.1 % reported one condition, 14.6 % reported two, and 13.6 % reported greater than two. The most common health conditions were depression (17.1 %), arthritis (15.1 %), anxiety (13.6 %), hypertension (13.1 %), and diabetes (9.0 %). Cardiovascular conditions were reported less frequently: heart attack or heart disease (2.5 %) and stroke (0.5 %). No respondents reported having kidney disease or other renal conditions.

**Table 1:** Demographic characteristics

Demographic characteristics	Statistics
	(N = 199)
Age (mean, SD)	46.3, 13.8
Gender (n, %)	
Female	109 (54.8 %)
Male	90 (45.2 %)
Ethnicity* (n, %)	
White	107 (53.8 %)
Black	24 (12.1 %)
Asian	52 (26.1 %)
Latin American	6 (3.0 %)
Other/Multiple	10 (5.0 %)
Marital status† (n, %)	
Single	109 (54.8 %)
Married	51 (25.6 %)
Other	39 (19.6 %)
Employment status‡, (n, %)	
Full-time work	65 (32.7 %)
Part-time work	57 (28.6 %)
Student	21 (10.6 %)
Retired	23 (11.6 %)
Unemployed	16 (8.0 %)
Other	17 (8.5 %)
Education Level (n, %)	
Completed University Degree	125 (62.8 %)
Did not Complete University Degree	74 (37.2 %)

*Other includes participants who reported multiple categories (n = 9) and participants who reported “other” (n = 1)

†Other includes divorced (n = 22), separated (n = 7), widowed (n = 8), and “other” (n = 2)

‡Other includes homemaker (n = 4), disabled (n = 7), and “other” (n = 6)

### Health state utilities

**Fig. 1:**
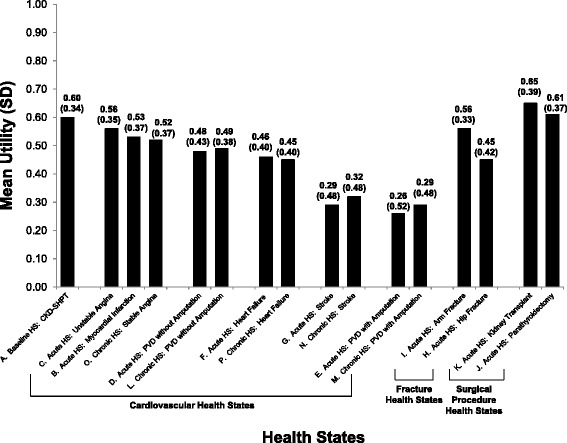
Time Trade-Off* Utility Scores. *TTO utility scores are on a scale anchored with 0 representing dead and 1 representing full health. HS = health state; TTO = time trade-off; PVD = peripheral vascular disease; CKD = chronic kidney disease; SHPT = secondary hyperparathyroidism. All 199 participants rated health state A. Mean utilities for other health states are based on evaluations from subsets of 130 to 135 participants. The precise number of participants who rated each health state is presented in Table 2. In this figure, acute cardiovascular health states are presented (grouped with their corresponding chronic states) in descending order from highest utility to lowest utility. The fracture and surgical procedure health states are also ordered from highest utility to lowest utility

Results of the introductory health state ranking task are presented in Table 2, and TTO utilities are presented in Fig. 1. Health state A was valued by all 199 participants who completed the interview, while every other health state was valued by approximately two-thirds of the sample (n = 130 to 135). The basic health state (A) describing CKD and SHPT on dialysis without additional clinical events had a mean utility of 0.60. Compared with health state A, the health states describing surgical interventions had higher mean utilities of 0.61 (J, parathyroidectomy) and 0.65 (K, kidney transplant).

**Table 2:** Health state ranking

Health state	N	Mean ranking*	SD	Range
A. Basic HS: CKD and SHPT	199	2.0	0.6	1.0–3.0
B. Acute HS: Myocardial Infarction	130	5.7	1.9	2.0–11.0
C. Acute HS: Unstable Angina	133	4.7	1.6	2.0–9.0
D. Acute HS: PVD without amputation	135	6.1	1.8	2.0–11.0
E. Acute HS: PVD with amputation	130	9.6	1.6	3.0–11.0
F. Acute HS: Heart Failure	133	7.5	2.0	4.0–11.0
G. Acute HS: Stroke	135	9.5	1.6	2.0–11.0
H. Acute HS: Hip Fracture	130	6.3	2.0	3.0–11.0
I. Acute HS: Arm Fracture	133	3.8	1.6	2.0–9.0
J. Acute HS: Parathyroidectomy	135	2.3	1.8	1.0–11.0
K. Acute HS: Kidney Transplant	130	1.6	1.7	1.0–11.0
L. Chronic HS: PVD without amputation	133	6.6	2.0	2.0–10.0
M. Chronic HS: PVD with amputation	135	9.6	1.6	4.0–11.0
N. Chronic HS: Stroke	130	9.1	1.7	3.0–11.0
O. Chronic HS: Stable Angina	133	6.3	1.9	2.0–11.0
P. Chronic HS: Heart Failure	135	7.3	2.1	3.0–11.0

*A lower mean ranking indicates that a health state was more preferable, while a higher mean ranking indicates that a health state was less preferable

HS = health state; PVD = peripheral vascular disease; CKD = chronic kidney disease; SHPT = secondary hyperparathyroidism

All other health states had lower mean utilities than health state A. Utilities for health states including acute cardiovascular events (B–G) ranged from 0.26 (E, PVD with amputation) to 0.56 (C, unstable angina). Utilities for chronic health states following a cardiovascular event (L–P) ranged from 0.29 (M, PVD with amputation) to 0.52 (O, stable angina). Health state H (hip fracture) had a utility of 0.45, while health state I (arm fracture) had a utility of 0.56.

There were no statistically significant differences in health state utilities between younger (n = 96) and older (n = 103) participants categorized based on a median split (median age = 48); male (n = 90) and female (n = 109) participants; or between participants who considered themselves religious (n = 103) and those who did not (n = 96).

### Disutilities associated with major clinical events

The disutility associated with the addition of each clinical event to health state A was computed by subtracting the utility of each event health state from the utility of health state A (Table 3). The event health states were identical to health state A other than the addition of the event. Therefore, any difference in utility score between health state A and the other health states represents the impact of the event on respondent preference. The two surgical events were associated with utilities higher than the utility for health state A, with mean differences of 0.02 for parathyroidectomy (J) and 0.06 for kidney transplant (K), indicating that the addition of these two events resulted in better perceived health-related quality of life.

**Table 3:** Differences in utility scores associated with major clinical events

Health State	N	Mean difference score*	SD	T value	P value
B-A. Acute HS: Myocardial Infarction	130	−0.06	0.13	−5.75	<0.0001
C-A. Acute HS: Unstable Angina	133	−0.05	0.10	−5.62	<0.0001
D-A. Acute HS: PVD without amputation	135	−0.11	0.22	−5.84	<0.0001
E-A. Acute HS: PVD with amputation	130	−0.33	0.39	−9.76	<0.0001
F-A. Acute HS: Heart Failure	133	−0.14	0.25	−6.51	<0.0001
G-A. Acute HS: Stroke	135	−0.30	0.35	−9.96	<0.0001
H-A. Acute HS: Hip Fracture	130	−0.14	0.22	−7.18	<0.0001
I-A. Acute HS: Arm Fracture	133	−0.04	0.09	−5.44	<0.0001
J-A. Acute HS: Parathyroidectomy	135	0.02	0.15	1.27	0.21
K-A. Acute HS: Kidney Transplant	130	0.06	0.25	2.73	0.0072
L-A. Chronic HS: PVD without amputation	133	−0.12	0.22	−6.25	<0.0001
M-A. Chronic HS: PVD with amputation	135	−0.30	0.32	−10.72	<0.0001
N-A. Chronic HS: Stroke	130	−0.27	0.35	−8.69	<0.0001
O-A. Chronic HS: Stable Angina	133	−0.09	0.17	−5.96	<0.0001
P-A. Chronic HS: Heart Failure	135	−0.14	0.20	−7.86	<0.0001

*This difference score was computed for each participant by subtracting the value of health state A from the value of the other health state

HS = health state; PVD = peripheral vascular disease

All other events were associated with disutilities (i.e., lower utility) ranging from a mean of −0.04 to −0.33. Mean disutilities for acute cardiovascular events (B–G) ranged from −0.05 (C, unstable angina) to −0.33 (E, PVD with amputation), while mean disutilities for chronic health states following a cardiovascular event (L–P) ranged from −0.09 (O, stable angina) to −0.30 (M, PVD with amputation). Of the two fracture health states, hip fracture (H) was associated with a larger mean disutility (−0.14) than arm fracture (I; −0.04). Differences between the utility of health state A and utilities of all cardiovascular and fracture health states (B–I, L–P) were statistically significant (p < 0.0001) (Table 3).

Among selected event health states that were conceptually comparable to each other, the following pairwise comparisons were statistically significant (Table 3): hip fracture (H) and arm fracture (I) (utility difference = 0.08); PVD with and without amputation, acute (D vs. E; utility difference = 0.19) and chronic health states (L vs. M; utility difference = 0.16); myocardial infarction and unstable angina (B vs. C; utility difference = 0.03).

## Discussion

In the current study cardiovascular events and fractures were associated with substantial disutility in the context of SHPT and CKD on dialysis. It has been suggested that differences among health state utilities of at least 0.05 can be considered clinically important [34]. The disutilities of all cardiovascular events and hip fracture met or exceeded this threshold, indicating that cardiovascular events and major fractures have an important impact on utility in the context of CKD and SHPT on dialysis. In addition, the utilities of all cardiovascular and fracture health states were significantly lower than the utility of CKD and SHPT without these added complications. In contrast, the two health states describing surgical interventions (J and K) were associated with higher utility values than the basic CKD and SHPT health state (A) due to the perceived improvements that could result from these surgeries. While the difference associated with parathyroidectomy was relatively small (+0.02), kidney transplant was associated with a more substantial utility increase (+0.06) over the basic health state. In light of these results, it is recommended that researchers conducting cost-utility models of treatment for CKD and SHPT on dialysis consider incorporating the utility differences associated with these important clinical events.

A unique contribution of the current study is the identification of disutilities associated with cardiovascular events and fractures in the context of CKD and SHPT. Previous cost-utility analyses of treatments for CKD and SHPT have used utilities for cardiovascular events and fractures from outside of the context of CKD due to the lack of utility data available for these events in this population [30–33]. However, the literature for cardiovascular events is highly variable [58], making it difficult to select the appropriate values for use in a model focusing on treatment of CKD. In addition, the majority of utilities for fractures have come from utility estimates in osteoporosis, which may not be relevant to patients with CKD [30–33]. Current health states were drafted based on clinicians’ descriptions of cardiovascular and fracture events specifically in the CKD context, and the impact of these events on utility were rated in this context. Therefore, current utility values may be uniquely appropriate for use in models focused on treatment for CKD and SHPT with dialysis.

While the current cardiovascular and fracture utilities are new in the CKD context, previous studies have reported utility values for CKD itself, as well as for patients who have received kidney transplants. The utility score of 0.60 for the health state representing CKD and SHPT on dialysis (A) is within the range of utilities for this type of patient obtained via a range of methods in previous studies. For example, a review found that TTO utilities for patients on hemodialysis across multiple studies ranged from 0.42 to 0.73 with a mean of 0.61, and utilities derived from the EQ-5D ranged from 0.44 to 0.62 with a mean of 0.56 [36]. Similar to results of the current study, this review reported that kidney transplant was associated with higher TTO (mean = 0.78) and EQ-5D (mean = 0.81) utilities. Another review reported average TTO utility means of 0.70 across studies for patients on dialysis, 0.82 for patients with kidney transplant, and an EQ-5D range from 0.44 to 0.71 for patients on dialysis [37]. The utility values for kidney transplant were higher in both of these reviews than in the current study, which is not unexpected since the utilities are representing different health concepts. These previous studies reported utilities of patients who had previously undergone a transplant and were living in a post-transplant state at the time of utility assessment. In contrast, the kidney transplant health state valued in the current study represented a year that included both the surgical procedure and the subsequent quality of life improvements. While the benefits of the surgery would lead to a greater utility value, the procedure itself was likely viewed as a negative event by most respondents, which would attenuate the utility gain associated with this health state. In sum, the previously published values represent a post-transplant health state, while the current utility of health state K represents a year including the transplant as well as subsequent improvements.

The TTO interviews in the current study were conducted with a one-year time horizon, and this methodology has yielded utility values with logical differences among health states (differences between health states were considered logical if the difference was in the expected direction, such as PVD with amputation having a lower utility than PVD without amputation). In TTO procedures, the duration of time spent in the health state being rated (i.e., the time horizon) is an important component of the task, and this time horizon varies across studies. Longer time horizons, most commonly 10 years, are used more frequently, but time horizons as short as one or two years have been shown to be useful in several previous TTO studies [59–68]. In the current study, the one-year time horizon was selected because a primary goal was to quantify the utility impact of acute events. Whereas this acute impact could be obscured if presented as a brief event within a 10-year time horizon, respondents clearly viewed the acute events as important when considered within the context of a single year. Therefore, the relatively brief time horizon seems to be an effective approach for quantifying the utility impact of acute events.

The time horizon does have implications for the eventual use of the utility scores. The health states each described a single hypothetical year of life, often involving a path or sequence of events such as a fracture followed by a gradual recovery. Consistent with the one-year paths described in the health states, they were rated in a TTO task with a one-year time horizon. Therefore, the utilities derived from this study represent valuations for hypothetical paths through one year of life, and can therefore be used in a cost utility model as representing one year. For example, the disutility of −0.06 associated with a myocardial infarction represents utility decrement across a single year. This disutility value would likely be different if it were representing the impact of myocardial infarction on a longer or shorter period of time. Consequently, when using these values in a cost-utility model, researchers need to be mindful of the timeframe, and the current disutilities should be applied to one-year time periods.

Modelers who intend to use the current values should also be aware of the conceptual difference between the acute and chronic health states. The acute health states represent a year that includes the acute event, and the corresponding disutility represents the utility decrement across the full year in which the event occurs. The chronic health states represent a post-event year, which is a year following an event, but not including the event itself. As with the acute health states, it is recommended that disutilities associated with the chronic health states be applied to a full year when used in a cost-utility model. This one-year time horizon allows results to be conceptualized in terms of the impact of cardiovascular and fracture events on a QALY. For example, the difference between health states A and G represents the QALY decrement associated with an acute stroke in the context of CKD and SHPT. The utilities reported here can also be used by modelers to represent trajectories of health-related quality of life in the context of CKD with SHPT. For instance, a patient might be in health state A for one year (utility = 0.60), suffer a stroke in year 2 (acute health state G; utility = 0.29), and then transition to chronic health state N representing the chronic impact of stroke (utility = 0.32) in year 3. The total (undiscounted) number of QALYs for the three-year period would be 1.21, compared with 1.8 for a patient without the stroke (i.e., 0.60*3).

The utility scores estimated in this study were derived from members of the general population, rather than patients with the relevant medical conditions, in order to approximate the societal viewpoint. This general population approach is consistent with guidance provided by health technology assessment agencies in many countries [53–56]. An advantage of a general population sample is that, because of the widespread use of utilities derived from general population valuations, scores derived via this approach are comparable to general population valuations of other health states. There are, of course, tradeoffs between scores obtained from the general population and scores obtained from patients who may have experienced some of the relevant health states. Patients may have greater insight into the content of health state descriptions, and therefore, patient-based utility valuations may be grounded in a better understanding of the health states. In this study, a concerted effort was made to draft health state descriptions that accurately represented the experience of patients with SHPT and CKD requiring dialysis, which may attenuate some of this concern. Nonetheless, it would be useful in future studies to compare general population and patient scores for these health states. In the meantime, the scores presented here may be used by modelers to estimate QALYs for major clinical events in the context of SHPT and CKD requiring dialysis.

Although this study yielded logical utilities (i.e., with relationships among health states in the expected directions), several aspects of the study design suggest that the results should be interpreted with appropriate caution. For example, utility assessment involving hypothetical health states is limited by the accuracy and level of detail in the health state descriptions. Therefore, utility scores derived via these methods may differ from patients’ ratings of their own health. However, identifying utilities associated with major clinical events directly from patients rating their own health would be impractical for two reasons. First, it would be difficult to identify patients and obtain utility values at the time the cardiovascular events or fractures occur. Second, even if a patient provides a utility at the time of the event, it may not be feasible to isolate the impact of a cardiovascular event or fracture on utility, separate from the impact of CKD and SHPT. In contrast, the hypothetical health state vignette approach used in the current study (which could be completed by either patients or general population respondents) is well-suited for isolating the impact on utility of specific medical conditions, health-related events, or treatments.

Another limitation of the current study is that the health states specified the names of the disease and medical events that were described. Some research has suggested that including the disease label in a health state can affect respondents’ valuations, while other studies have reported situations when the label did not affect valuations [69–71]. Although some researchers recommend omitting the disease label from health states, others prefer to label the medical condition as it might minimize misunderstanding of the health state. Furthermore, inclusion of the label more closely mirrors the patient experience, as patients clearly know the name of their condition. The current study included a complex set of health states representing a diverse range of medical concepts, including kidney disease, SHPT, dialysis, cardiovascular disease, bone-related complications, two types of surgery, and acute versus chronic effects. Therefore, each medical issue was explicitly named to make it easier for respondents to comprehend and differentiate between the health states. Additionally, some of the cardiovascular states were similar to each other (e.g., myocardial infarction and unstable angina), and the labels were necessary to ensure that participants did not become confused during the TTO task. However, it should be acknowledged that inclusion of the labels could have influenced utility scores.

An additional limitation relates to negative utility values. The health states in this study describe severe medical conditions, and all health states were rated by at least one respondent as worse than dead. With TTO methods, health states worse than dead are rated in a slightly different procedure than states with positive scores, meaning that worse than dead valuations are not measured on the same scale as states with positive scores [49]. While this limitation is relevant to all utility assessment, it is a more important issue when assessing severe health states that are likely to elicit more negative values.

Sample selection may be considered another limitation. Although efforts were made to ensure that the sample was balanced with regard to key demographic variables such as age, gender, ethnic/racial background, and employment status, the sample was not recruited to be nationally representative. Furthermore, participants reported relatively high rates of some clinical conditions (e.g., depression, arthritis, anxiety). Therefore, generalizability to the Canadian population or the population of other countries is not known, although there is no reason to believe that current values would be systematically different from a nationally representative sample.

Despite limitations, the current study is a step toward more thorough and accurate modeling of treatment for patients with CKD and SHPT. The current utility differences associated with cardiovascular events and fractures may be used to represent these debilitating events in cost-utility analyses specifically focusing on patients with CKD and SHPT on dialysis. Future research may examine whether current values derived from health state descriptions are consistent with utilities derived from actual patients who are experiencing these important clinical events.

## Additional file

Additional file 1: Appendix A. Full text of all 16 health states.
