# Supplementary material for: Health state utilities associated with major clinical events in the context of secondary hyperparathyroidism and chronic kidney disease requiring dialysis
Source: Health Qual Life Outcomes. 2015 Jun 30;13:90. doi: 10.1186/s12955-015-0266-9 (PMC4487205; doi:10.1186/s12955-015-0266-9)
Supplement: Additional file 1: Appendix A. — Full text of all 16 health states. [file 12955_2015_266_MOESM1_ESM.docx]

APPENDIX A. FULL TEXT OF ALL 16 HEALTH STATES

HEALTH STATE A:

Chronic kidney disease with secondary hyperparathyroidism

- You have chronic kidney disease with loss in kidney function.
- As a result, you have higher levels of calcium and phosphorus in your blood than someone without the disease. This is known as secondary hyperparathyroidism.
- Because of this condition, you have the symptoms described below.

Symptoms

- You often have:
- Aching pain in your bones, joints, and muscles.
- Itching that is very irritating.
- Trouble sleeping (you wake up several times each night).
- Fatigue (you feel tired).

Impact

- You have some difficulty performing household activities such as cooking and cleaning.
- You are able to work, but cannot do any heavy lifting.
- You find it difficult to stand for extended periods of time.
- You are able to socialize for limited periods of time, but you find it difficult to participate in any sports or physical activities.

Dialysis

- You visit the hospital 3 times each week for 3-4 hours for a dialysis treatment.
- In dialysis, you have a tube inserted into a vein (most likely in your arm), which is then attached to a machine that filters and cleans your blood.

HEALTH STATE B:

Same as Health State A

- Chronic kidney disease and secondary hyperparathyroidism.
- You also have the health issues described below.

Heart Attack

- Early in the year, you have a myocardial infarction, which is also called a heart attack. A heart attack occurs when the supply of blood to the heart is suddenly blocked.
- This can cause damage to the heart muscle.
- Your symptoms include:
- An uncomfortable pressure and crushing pain in the center of your chest that last a few minutes, and you have pain in your arm.
- Shortness of breath.
- A cold sweat, nausea, and you feel lightheaded.
- You are taken to the hospital.

After the Heart Attack

- You spend one week in the hospital, after which you return home.
- After returning home, you no longer experience the initial heart attack symptoms described above.
- You feel tired and fatigued for a few months.

HEALTH STATE C:

Same as Health State A

- Chronic kidney disease and secondary hyperparathyroidism.
- You also have the health issues described below.

Unstable Angina

- Early in the year, you experience a heart condition called unstable angina.
- Unstable angina occurs when the blood supply to the heart muscles is temporarily restricted. This can occur very suddenly.
- Damage to your heart muscle is less severe than damage from a heart attack.
- Your symptoms include:
- An uncomfortable pressure and crushing pain in the center of your chest that lasts a few minutes, and you have pain in your arm.
- Shortness of breath.
- A cold sweat, nausea, and you feel lightheaded.
- You are taken to the hospital.

After the Unstable Angina

- You spend two days in the hospital, after which you return home.
- After returning home, you no longer experience the symptoms of unstable angina described above.
- You feel tired and fatigued for a few months.

HEALTH STATE D:

Same as Health State A

- Chronic kidney disease and secondary hyperparathyroidism.
- You also have the health issues described below.

Peripheral Vascular Disease

- Early in the year, you are diagnosed with peripheral vascular disease, which involves several blockages in the blood vessels in your lower leg.
- You have pain in your lower leg after walking 5-10 minutes.
- You have poor circulation to your foot, and your foot is cold and numb.
- You find it difficult to walk for more than 100 meters due to the pain.
- Because the blockage has affected blood flow to your foot, a small amount of skin on your foot has turned black.

After Peripheral Vascular Disease

- You visit the hospital to receive a treatment to remove the dead tissue. This will require a general anesthetic, and you will not be awake for the procedure.
- After the treatment, you continue to have pain in your leg after walking.

HEALTH STATE E:

Same as Health State A

- Chronic kidney disease and secondary hyperparathyroidism.
- You also have the health issues described below.

Peripheral Vascular Disease

- Early in the year, you are diagnosed with peripheral vascular disease, which involves several blockages in the blood vessels in your lower leg.
- You have pain in your lower leg after walking 5-10 minutes.
- You have poor circulation to your foot, and your foot is cold and numb.
- You find it difficult to walk for more than 100 meters due to the pain.
- Because the blockage has affected blood flow to your foot, a small amount of skin on your foot has turned black.

After Peripheral Vascular Disease: Amputation

- You visit the hospital to receive treatment to remove the dead tissue, but the treatment has not been successful.
- Your leg has to be amputated below the knee.
- After returning home from the hospital, you no longer have symptoms of peripheral vascular disease.
- You have to use a wheelchair for mobility.

HEALTH STATE F:

Same as Health State A

- Chronic kidney disease and secondary hyperparathyroidism.
- You also have the health issues described below.

Heart Failure

- Early in the year, you suffer from heart failure which means that your heart is unable to pump enough blood throughout your body.
- You have shortness of breath, chest pain, heart palpitations and you are admitted to the hospital.

After Heart Failure

- You spend one week in the hospital, after which you return home.
- After returning home, you continue to suffer from tiredness, fatigue, and shortness of breath.

HEALTH STATE G:

Same as Health State A

- Chronic kidney disease and secondary hyperparathyroidism.
- You also have the health issues described below.

Stroke

- Early in the year, you have a stroke, which is a blockage in a blood vessel in the brain.
- This can cause damage to your brain.
- This causes the following symptoms that last for a few hours:
- Weakness of your face, arm, and leg on one side of your body.
- **Difficulty walking**, dizziness, and loss of balance.
- Confusion, difficulty speaking and understanding others speaking.
- A severe headache.
- You have to go to the hospital. After you have been stabilized and recovered, you receive physiotherapy in the hospital for 3 to 4 weeks.

Symptoms After the Stroke

- You have numbness and weakness of your face, arm, and leg on one side of your body.
- Your speech is occasionally slurred, and as a result, you sometimes find it difficult to communicate.

Impact After the Stroke

- You struggle to perform complex tasks due to difficulty with coordination.
- When walking, you require walking aids (such as a cane or a wheelchair).
- You struggle to walk long distances of more than 100 meters, and you often get dizzy when walking.

HEALTH STATE H:

Same as Health State A

- Chronic kidney disease and secondary hyperparathyroidism.
- You also have the health issues described below.

Hip Fracture

- You have weaker bones due to your condition. Early in the year, you suffer a hip fracture, which causes the following short term symptoms:
- Extreme pain in your hip.
- An inability to lift, move, or rotate your hip.
- Inability to stand or put weight on your leg.
- You have to go to the hospital and have an operation to help the hip fracture to mend.

After the Fracture

- You have to stay in the hospital for up to a week.
- After you return home, you receive physiotherapy.
- You have difficulty walking, bathing, and dressing for up to 6 months.

HEALTH STATE I:

Same as Health State A

- Chronic kidney disease and secondary hyperparathyroidism.
- You also have the health issues described below.

Arm Fracture

- You have weaker bones due to your condition. Early in the year, you suffer an arm fracture which causes:
- Extreme pain in your arm.
- You are unable to move your arm or lift any weight with the hand of your affected arm.

After the Arm Fracture

- You have a cast on your arm for a few weeks while it heals.
- The pain will lessen during this time, until the arm has healed about a month or two after the fracture.

HEALTH STATE J:

Same as Health State A

- Chronic kidney disease and secondary hyperparathyroidism.
- You also have the health issues described below.

Operation: Parathyroidectomy

- Early in the year, you have to have an operation:
- This operation is performed to treat your symptoms of secondary hyperparathyroidism (SHPT).
- This operation involves surgery to your neck to remove the parathyroid glands.
- You will undergo a general anesthetic and you will not be awake during the procedure.

After the Operation

- For the first month after your surgery, your symptoms of SHPT are still present. These include:
- Aching pain in your bones, joints, and muscles.
- Itching that is very irritating.
- Trouble sleeping (you wake up several times each night).
- Fatigue (you feel tired).
- After the first month or two, your symptoms of SHPT are significantly improved.
- You continue to visit the hospital 3 times each week for 3-4 hours for dialysis treatment.

HEALTH STATE K:

Same as Health State A

- Chronic kidney disease and secondary hyperparathyroidism.
- You also have the health issues described below.

Kidney Transplant

- Early in the year, you undergo a kidney transplant operation.
- You receive a general anesthetic during the surgery and you are not awake during the operation.
- You will remain in the hospital for a week following the operation.

After the Transplant

- For the first 6 weeks you cannot perform strenuous activities such as driving, cleaning the house, lawn mowing, and lifting.
- For the first few weeks after the operation, you need to visit the hospital 2 or 3 times each week to check that the transplanted kidney is working as it should.
- After a few months, your health and symptoms significantly improve because your new kidney is working.
- You need to take anti-rejection medication every day to prevent your body from rejecting the new kidney, and you still see a kidney specialist regularly.
- You no longer need dialysis treatment.

HEALTH STATE L:

Same as Health State A

- Chronic kidney disease and secondary hyperparathyroidism.
- You also have the health issues described below.

Peripheral Vascular Disease in a Prior Year

- Prior to this year, you were diagnosed with peripheral vascular disease, which involves several blockages in the blood vessels in your lower leg.

Ongoing Symptoms

- You have pain in your lower leg that comes on after walking and lasts for about 5-10 minutes.
- Your feet often feel numb and cold.

Impact

- You find it difficult to walk for more than 100 meters at a time.
- For distances greater than 100 meters, you require walking aids (such as a cane or a wheelchair) to remove the strain on your legs and to reduce the pain.
- You experience discomfort even when resting, especially if your leg is raised.

HEALTH STATE M:

Same as Health State A

- Chronic kidney disease and secondary hyperparathyroidism.
- You also have the health issues described below.

Peripheral Vascular Disease in a Prior Year: Amputation

- Prior to this year, you were diagnosed with peripheral vascular disease, which involves several blockages in the blood vessels in your lower leg.
- This caused symptoms including pain in your lower leg, numbness in your foot, and difficulty walking for more than 100 meters at a time.
- As a result of this condition, you had to have your leg amputated below the knee.

Impact

- As a result of the amputation, you are unable to walk, and you use a wheelchair.
- You find it difficult to perform activities such as cooking, cleaning, dressing, and washing yourself.

HEALTH STATE N:

Same as Health State A

- Chronic kidney disease and secondary hyperparathyroidism.
- You also have the health issues described below.

Stroke in a Prior Year

- Prior to this year, you experienced a stroke, which is a blockage in a blood vessel in the brain.
- This can cause damage to your brain.
- The continuing effects on your life include the following symptoms and impact.

Ongoing Symptoms

- You have numbness and weakness of your face, arm, and leg, on one side of your body.
- Your speech is occasionally slurred, and as a result, you sometimes find it difficult to communicate.

Ongoing Impact

- You struggle to perform complex tasks due to difficulty with coordination.
- When walking, you require walking aids (such as a cane or a wheelchair).
- You struggle to walk long distances of more than 100 meters, and you often get dizzy when walking

HEALTH STATE O:

Same as Health State A

- Chronic kidney disease and secondary hyperparathyroidism.
- You also have the health issues described below.

Stable Angina

- Prior to this year, you experienced a heart condition called unstable angina, and you received medical treatment. Unstable angina occurs when the blood supply to the heart muscles is temporarily restricted. This can occur very suddenly.
- Now you are diagnosed with a heart condition called stable angina. These symptoms usually occur after activities.
- Symptoms of stable angina are not as sudden as those of unstable angina.

Symptoms

- After activities such as climbing stairs, you experience:
- An uncomfortable pressure, squeezing, fullness, or pain in the centre of your chest that lasts for a few minutes after stopping the activity.
- Shortness of breath.
- Cold sweat, nausea, or light-headedness.

Impact

- You find it difficult to perform strenuous activities due to the pain and shortness of breath.
- You require frequent breaks when walking long distances of more than 100 meters.

HEALTH STATE P:

Same as Health State A

- Chronic kidney disease and secondary hyperparathyroidism.
- You also have the health issues described below.

Heart Failure in a Prior Year

- Prior to this year, you experienced a heart failure episode and had medical treatment.
- Your heart was unable to pump blood effectively throughout your body.
- The continuing effects of this condition on your life include the following symptoms and impact.

Symptoms

- You continue to suffer from tiredness, fatigue, shortness of breath, swollen ankles, and have difficulty sleeping.

Impact

- You find it difficult to walk long distances of more than 100 meters or perform other strenuous activities, and you quickly feel out of breath.
